# Oncophone20 study: Patients’ perception of telemedicine in the COVID‐19 pandemic during follow‐up visits for gynecological and breast cancers

**DOI:** 10.1002/ijgo.13825

**Published:** 2021-07-29

**Authors:** Elisa Picardo, Maria G. Baù, Caterina Anatrone, Aurelia Mondino, Alessandra Surace, Federica Gallo, Saverio Danese, Marco Mitidieri

**Affiliations:** ^1^ Department of Gynecology and Obstetrics Sant'Anna Hospital AOU Citta della Salute e della Scienza di Torino Torino Italy; ^2^ Surgical Sciences University of Turin Torino Italy; ^3^ Epidemiology Unit Health Direction, Local Health Authority 1 Cuneo Italy

**Keywords:** breast cancer, COVID‐19, follow‐up, gynecology oncology, telemedicine

## Abstract

**Objective:**

To analyze oncological patients’ perception of telemedicine during the COVID‐19 pandemic.

**Methods:**

A total of 345 women, of whom 267 experienced breast cancer and 78 experienced a gynecological cancer, were enrolled. Specific questionnaires about their experiences and feelings about telemedicine in the COVID‐19 era were collected.

**Results:**

In the breast group, “enhanced care” showed moderate positive perception (mean 4.40) among less‐educated women that was slightly lower among better‐educated women (mean 4.14) with a significant difference (*P* = 0.034). “satisfaction” had an opposite pattern: a mean of 3.99 for a lower level of education and 4.78 for a higher level of education, with a strong significant difference (*P* < 0.001). “privacy and discomfort” approached neutrality for less‐educated women, while for higher‐educated women the lower mean of 2.93 indicted a more positive perception (*P* = 0.007). In the pelvic group, younger women had a better perception towards telemedicine for “telemedicine as a substitution” (mean 3.68) compared to older women (mean 3.05). The privacy and discomfort subscale was in favor of better‐educated women (mean 2.57) compared to less‐educated women (mean 3.28; *P* = 0.042).

**Conclusion:**

Telemedicine was generally well accepted, not only among younger and higher‐educated women but also by women needing intensive care, in both cancer groups.

## Synopsis

Telemedicine represents a strategic tool for the control of COVID‐19 infection in patients, caregivers, and healthcare professionals and was generally well accepted.

## 1 INTRODUCTION

The COVID‐19 pandemic has increased the burden on healthcare systems worldwide. In order to ensure adequate clinical and psychological care, maintaining home confinement, patients were included in a multidisciplinary tele‐assistance network. Based on the results obtained during the pandemic, many experts of “integrated care” and “patient‐centered care” encouraged telemedicine as an elective strategy for medical follow‐up. The Communication of the European Commission COM(2008)689 entitled “Telemedicine for the benefit of patients, health‐care systems and society,” released by the European Commission, aimed to support States in the large‐scale implementation of telemedicine services through specific initiatives, such as: creating trust in telemedicine services; promoting their acceptance; bringing legal clarity; resolving technical issues; and facilitating market development. In many European countries, telemedicine is widespread and, in some cases, supported by regulatory interventions, strategic documents, and national projects.

The development of tools for telemedicine allows both the finding of new solutions to the traditional problems of medicine and the creating of new opportunities to improve health services through greater cooperation between different healthcare professionals and patients.

The aim of the present study was to analyze the continuous and multidisciplinary management of patients in follow‐up for gynecological cancer regarding the perception of the disease during the COVID‐19 pandemic, logistical improvement, and the level of care, trust, and resilience.

## 2 METHODS

### 2.1 Enrolment of patients and therapy

The research protocol was approved by the Ethics Committee of Città della Salute e della Scienza of Turin before the study began. All recruited patients gave informed consent at the beginning of the phone calls, and all patients were free to accept or refuse the interview without affecting the routine follow‐up visit. A total of 345 women were consecutively enrolled from the beginning of March 2020 to the end of May 2020: there were 267 women in the breast cancer group and 78 women in the pelvic cancer group.

According to the Italian guidelines (AIOM), a follow‐up visit after breast cancer consists of a clinical visit with the evaluation of hematologic exams (if they are required for any clinical reasons, that is metabolic risk assessment during aromatase inhibitor therapy) every 6 months and mammography once a year for the first 5 years. Other radiological examinations (breast and axilla ultrasounds, magnetic resonance imaging, positron emission tomography, and others) can be considered in case of high risk of recurrence or prosthesis. A follow‐up visit after cervical cancer consists of a clinical visit, colposcopy, and cytological examination (cervical smear test) every 3–6 months for the first 2 years and every 6 months for the next 3 years. A follow‐up visit after endometrial cancer consists of a clinical visit every 3–4 months for the first 3 years and every 6 months for the next 2 years. The type of therapy administered to each patient was available through hospital computer records: surgery, chemotherapy treatment, hormonotherapy, and radiotherapy for breast and pelvic cancer.

Due to COVID‐19 limitations, the study hospital decided to organize follow‐up visits differently from the beginning of the pandemic in March 2020. To prevent patients from coming to the hospital, the secretary notified the patients on the subsequent phone call from the oncologist of the replacement for the ambulatory follow‐up visits. During the phone call, the physician evaluated the health status and possible hematologic results of the patients by asking them direct questions. Patients with a history of previous gynecological cancer (age above 18 years) were enrolled in the study during routine follow‐up phone calls. The Service User Technology Acceptability Questionnaire (SUTAQ),^1^ reported in appendix 1, was administered by phone call after about 2 months after the follow‐up phone call. The mean estimated time to answer the questionnaire was 20 min. To evaluate the perception of the phone call for the follow‐up visit in patients with gynecological cancers, a survey was conducted through a self‐administered SUTAQ with 27 closed questions.^2^, ^3^, ^4^ The questionnaire was translated from English into Italian.^5^ All patients needed a good understanding of the Italian language in order to answer the questions. The questionnaire aimed to highlight patients’ perception about telemedicine. It consisted of questions about their telemedicine experience and feelings during the COVID‐19 pandemic. Specifically, it consisted of 22 items divided into six different subscales: “enhanced care”; “satisfaction”; “increased accessibility”; “telemedicine as a substitution” (TMS); “privacy and discomfort”; and “care personnel concerns” (CPC). The enhanced care subscale was concerned with questions about patients’ worries regarding their health status, their sense of active involvement, and enhanced care. The satisfaction subscale dealt with questions about patients’ satisfaction and their agreement on telemedicine. The increased accessibility subscale dealt with patients’ perception of saving time and their increased access to health care and health professionals. The TMS subscale was about patients’ concerns about their health status and a phone call as a substitute for regular care. The privacy and discomfort subscale involved patients’ worries about privacy and their perception of discomfort. The CPC subscale included questions about patients’ perception of continuity of care and considerations related to personnel involved in the service. The phrasing of the 22 items in the 6‐point Likert scale questionnaire was both positive and negative in order to reduce related bias. The final results of each subscale demonstrated the degree of average consensus (6 indicated strong agreement and 1 indicated strong disagreement). The enhanced care, satisfaction, increased accessibility, and TMS subscales indicated the degree of internal agreement (6 indicated strong agreement and 1 indicated strong disagreement), so a high value reflected a positive perception of telemedicine in these aspects. privacy and discomfort and CPC were inverted subscales, so a low value reflected a positive perception in these aspects. The intermediate value of 3.5 was considered as a point of neutrality for all subscales. A further five questions about level of education, type of job, and family composition were added to the questionnaire.

### 2.2 Statistical analyses

To compare the SUTAQ questionnaire results among the breast cancer group versus the pelvic cancer group, average values and standard deviations were calculated for each subscale, stratified by age group (≤55 years vs ≥56 years), level of education (middle school or below vs high school or above), and intensity of treatment (intensive care vs non‐intensive case). Actual comparisons were then performed using *t*‐tests. In the pelvic group, five different sites of cancer (endometrial, ovarian, vulvar, vaginal, and cervical cancer) were included, with deep differences in treatment.

For statistical analyses, the treatments were separated into intensive care and non‐intensive care: care intensity was defined “intensive care” if the patient received chemotherapy and/or radiotherapy after surgery, and defined as “non‐intensive” if the patient received hormonotherapy or only follow‐up after surgery.

To investigate the role of sociodemographic characteristics of patients towards their acceptance of telemedicine, a classical linear regression model was run. A total score summarizing the whole SUTAQ was found as the sum of the subscales reflecting a positive perception of telemedicine (enhanced care, satisfaction, increased accessibility, TMS) minus the subscales reflecting uncertainty about telemedicine (privacy and discomfort, CPC). No departure from normality was detected by visual inspection (histogram and Q‐Q plot). The total score of the SUTAQ was considered to be the outcome and the following dependent variables were included as regressors: age at follow‐up (continuous); marital status (single vs married or in a relationship); level of education (middle school or less vs high school or more), and occupational status (unemployed vs employed/students). The model was adjusted by cancer site (breast/pelvic).

Data analyses were performed using SAS version 9.4 (SAS Institute Inc, Cary, NC, USA).

## 3 RESULTS

A total of 346 women were enrolled: 267 in the breast cancer group and 79 in the pelvic cancer group. Women in the pelvic cancer group were younger (median age 56 years) and had a higher level of education (61.53% achieved a high school diploma or higher) than those in the breast cancer group (median age 67 years; 23.22% with a high level of education). The majority of patients in the pelvic cancer group were employed or students (87.34%) compared to 75.28% of women in the breast cancer group. In the pelvic cancer group, 70.89% of women were living with their partner compared to 37.08% in the breast cancer group.

Of the women enrolled with breast cancer, 18% received chemotherapy treatment, 71% received hormonotherapy, and 67% received radiotherapy. Of the women in the pelvic cancer group, 41.8% received chemotherapy treatment, 20.25% received radiotherapy, and 37.95% had only a follow‐up after surgery.

Table 1 compares the SUTAQ subscales between the breast cancer and pelvic cancer groups. The enhanced care, satisfaction, and increased accessibility subscales showed a mild agreement in both groups (mean values of approximately 4). While the differences between the enhanced care and increased accessibility groups were not significant, the satisfaction group approached statistical significance. For TMS, agreement was a bit lower, especially in the pelvic cancer group (3.90 for breast cancer and 3.33 for pelvic cancer) and the mean difference with the other group was statistically significant. These results are suggestive of a moderate positive perception of telemedicine for both groups. privacy and discomfort and CPC were below the point of neutrality for both groups, again suggesting a positive perception of telemedicine. In particular, privacy and discomfort showed a stronger perception in the pelvic cancer group than in the breast cancer group (mean values of 3.30 for breast cancer and 2.86 for pelvic cancer) and was statistically significant (*P* = 0.018). This is suggestive of a stronger positive perception in the pelvic group towards continuity of care and possible concerns about health operators.

**TABLE 1:** Comparison of SUTAQs: breast cancer group versus pelvic cancer group^a^

	Breast cancer group (*n* = 267)	Pelvic cancer group (*n* = 79)	*t*‐test comparison
SUTAQ subscale			
EC	4.43 ± 0.73	4.36 ± 1.31	0.651
ST	4.18 ± 1.03	4.51 ± 1.44	0.058
IA	4.08 ± 0.74	4.19 ± 1.20	0.447
TMS	3.90 ± 0.98	3.33 ± 1.25	**0.001**
PD	3.30 ± 0.96	2.86 ± 1.51	**0.018**
CPC	3.36 ± 1.34	3.08 ± 1.45	0.110

Note

Statistically significant differences between groups are in bold.

Abbreviations: CPC, care personnel concerns; EC, enhanced care; IA, increased accessibility; PD, privacy and discomfort; ST, satisfaction; SUTAQ, Service User Technology Acceptability Questionnaire; TMS, telemedicine as a substitution.

^a^ Values are given as mean ± SD.

Table 2 compares the SUTAQ subscales in the breast group by age, level of education, and intensity of care. A significant mean difference was observed for age, as the increased accessibility group showed moderate positive perception (mean 3.90) among women aged 55 years or below, while older women had a stronger positive perception (mean 4.13) with a significant difference (*P* = 0.031). Regarding level of education, significant differences were observed. The enhanced care subscale showed moderate positive perception among less‐educated women (mean 4.48) that was slightly lower among higher‐educated women (mean 4.23) with a significant difference (*P* = 0.017). Satisfaction and TMS showed an opposite pattern: for the satisfaction subscale, the mean was 3.99 for low level of education and 4.78 for high level of education, with a strong significant difference (*P* < 0.001). For TMS, the mean was 3.82 for low level of education and 4.18 for high level of education (*P* = 0.028). The privacy and discomfort subscale approached neutrality for low level of education, while for higher‐educated women it had a lower mean of 2.93, indicating a more positive perception (*P* = 0.001). No significant differences were detected between intensive and non‐intensive treatment.

**TABLE 2:** Comparisons of SUTAQ by age group, level of education, and intensity of care in the breast cancer group^a^

	Age ≤55 years (*n* = 61)	Age ≥56 years (*n* = 206)	*t*‐test comparison
SUTAQ subscale			*P* value
EC	4.51 ± 0.67	4.40 ± 0.75	0.288
ST	4.12 ± 1.02	4.19 ± 1.03	0.623
IA	3.90 ± 0.71	4.13 ± 0.74	**0.031**
TMS	3.94 ± 1.01	3.89 ± 0.98	0.753
PD	3.31 ± 0.93	3.29 ± 0.97	0.922
CPC	3.51 ± 1.13	3.32 ± 1.39	0.341

	Education ≤middle school (*n* = 205)	Education ≥high school (*n* = 62)	*t*‐test comparison
SUTAQ subscale			
EC	4.48 ± 0.73	4.23 ± 0.72	**0.017**
ST	3.99 ± 0.90	4.78 ± 1.18	**<0.001**
IA	4.07 ± 0.68	4.11 ± 0.92	0.747
TMS	3.82 ± 0.90	4.18 ± 1.17	**0.028**
PD	3.41 ± 0.82	2.93 ± 1.27	**0.001**
CPC	3.42 ± 1.31	3.19 ± 1.42	0.252

	Intensive care (*n* = 149)	Non intensive care^b^ (*n* = 98)	*t*‐test comparison
SUTAQ subscale			
EC	4.42 ± 0.80	4.38ׅ ± 0.65	0.691
ST	4.14 ± 1.09	4.24 ± 0.94	0.437
IA	4.05 ± 0.74	4.11 ± 0.71	0.521
TMS	3.88 ± 1.04	3.92 ± 0.92	0.802
PD	3.28 ± 0.98	3.40 ± 0.89	0.232
CPC	3.39 ± 1.36	3.38 ± 1.30	0.962

Note

Statistically significant differences between groups are in bold.

Abbreviations: CPC, care personnel concerns; EC, enhanced care; IA, increased accessibility; PD, privacy and discomfort; ST, satisfaction; SUTAQ, Service User Technology Acceptability Questionnaire; TMS, telemedicine as a substitution.

^a^ Values are given as mean ± SD.

^b^ Intensity of treatment was not determined for 20 women due to incomplete data.

Table 3 compares the SUTAQ subscales in the pelvic group by age, level of education, and intensity of care. For TMS, the positive perception towards telemedicine was higher among younger women (mean 3.68) compared to older women (mean 3.05) with a significant difference (*P* = 0.025). The privacy and discomfort subscale was in favor of higher‐educated women (mean 2.57) compared to lesser‐educated women (mean 3.28; *P* = 0.042). No significant differences were detected between intensive and non‐intensive treatment. In fact, the enhanced care, satisfaction, increased accessibility, and privacy and discomfort subscales reached good responsiveness towards telemedicine, irrespective of care level.

**TABLE 3:** Comparison of SUTAQ by age group, level of education, and intensity of care in the pelvic cancer group^a^

	Age ≤55 years (*n* = 35)	Age ≥56 years (*n* = 44)	*t*‐test comparison
SUTAQ subscale			*P* value
EC	4.52 ± 1.20	4.22 ± 1.39	0.310
ST	4.72 ± 1.37	4.34 ± 1.79	0.242
IA	4.43 ± 1.17	3.99 ± 1.19	0.104
TMS	3.68 ± 1.21	3.05 ± 1.23	**0.025**
PD	2.83 ± 1.45	2.89 ± 1.56	0.854
CPC	2.88 ± 1.53	3.25 ± 1.36	0.256

	Education ≤middle school (*n* = 30)	Education ≥high school^b^ (*n* = 48)	*t*‐test comparison
SUTAQ subscale			
EC	4.53 ± 1.20	4.22 ± 1.37	0.326
ST	4.73 ± 1.40	4.34 ± 1.45	0.243
IA	4.20 ± 1.11	4.18 ± 1.27	0.935
TMS	3.50 ± 1.21	3.21 ± 1.28	0.322
PD	3.28 ± 1.71	2.57 ± 1.31	**0.042**
CPC	3.07 ± 1.27	3.04 ± 1.53	0.940

	Intensive care (*n* = 42)	Non‐intensive care (*n* = 37)	*t*‐test comparison
SUTAQ subscale			
EC	4.24 ± 1.43	4.49 ± 1.15	0.394
ST	4.33 ± 1.55	4.71 ± 1.29	0.246
IA	4.05 ± 1.16	4.34 ± 1.24	0.283
TMS	3.17 ± 1.21	3.51 ± 1.29	0.233
PD	3.09 ± 1.60	2.61 ± 1.36	0.158
CPC	3.23 ± 1.47	2.92 ± 1.42	0.343

Note

Statistically significant differences between groups are in bold.

Abbreviations: CPC, care personnel concerns; EC, enhanced care; IA, increased accessibility; PD, privacy and discomfort; ST, satisfaction; SUTAQ, Service User Technology Acceptability Questionnaire; TMS, telemedicine as a substitution.

^a^ Values are given as mean ± SD.

^b^ Level of education was not determined for one woman due to incomplete data.

Table 4 shows the influence of certain sociodemographic factors on patients’ perception of telemedicine. Significant coefficients were estimated for level of education (*P* = 0.047) and occupational status (*P* < 0.001). These results highlight that higher‐educated women have a more positive approach toward telemedicine. Conversely, employed women/students seemed to be less satisfied with this service compared to unemployed women.

**TABLE 4:** Sociodemographic characteristics influencing acceptance of telemedicine

Sociodemographic factors	Parameter estimate (SE)	*t*‐statistic	*P* value
Age	0.02 (0.02)	0.95	0.341
Marital status (attached)	0.79 (0.47)	1.68	0.093
Level of education (≥high school)	1.02 (0.51)	1.99	**0.047**
Occupational status (employed/students)	−2.27 (0.54)	−4.14	**<0.001**

Note

Statistically significant coefficients are in bold.

Abbreviation: SE, standard error.

## 4 DISCUSSION

The WHO definition of telemedicine is “the delivery of health care services, where distance is a critical factor, by all health care professionals using information and communication technologies for the exchange of valid information for diagnosis, treatment and prevention of disease of injuries, research, and evaluation, and for the continuing education of health care providers, all in the interests of advancing the health of individuals and their communities”.^6^ Moreover, in most of the previous reviews, telemedicine was defined as the evaluation of patients by a healthcare professional by a written, audio, or video method.^7^, ^8^ COVID‐19 infection has swept quickly across countries since March 2020, causing a delay in many elective procedures among different medical specialties.^9^ In the study institution, activities such as follow‐up for oncological female cancers were paused for 1 month, to divert resources to COVID‐19 care. In Italy, central government cited a lockdown of all people except in special circumstances starting from March 9, 2020. One month after stopping these activities, realizing that patients with cancer needed to be cared for, follow‐up visits were performed by telephone and the Internet for the evaluation of hematologic and radiologic examinations. Telemedicine has been reported in the literature as a strong and long‐lasting pillar of oncology.^10^, ^11^ Since telemedicine had not been previously adopted at the study institution, available tools such as Android and IOS applications for chatting and videocalls as well as other artificial‐intelligence–based tools and software were not immediately available. Therefore, telephone social networks were used to begin the project. After 3 months, patient feedback about this experimental kind of telemedicine was evaluated to utilize telemedicine in the future regardless of the COVID‐19 pandemic. Telemedicine was a useful and convenient tool because the risk of infection of COVID‐19 in crowded outpatient clinic waiting rooms was reduced to a minimum.

The results of the present study show that patients with pelvic cancer were more satisfied with telemedicine compared to patients in the breast cancer group (approaching statistical significance, *P* = 0.058). Moreover, the breast cancer group reported telemedicine as comparable to a face‐to‐face appointment more than the pelvic cancer group, even though they were overall less satisfied (statistically significant, *P* = 0.001). It is possible that patients with pelvic cancer regard a gynecological examination as necessary for a follow‐up with pathology instead of other methods such as a telemedicine‐only approach. In the study population, patients with breast cancer are older and less educated than those with pelvic cancer, and this could explain the differences observed among the two groups. With regard to privacy and discomfort, there was a general acceptance of telemedicine as a substitute for a face‐to‐face visit. Among the pelvic cancer group, discomfort and lack of privacy were considered more important than in the breast cancer group (statistically significant, *P* = 0.018). In all groups, the perception reported about telemedicine was positive for all the items reported by the SUTAQ.^2^ Telemedicine represents a strategic tool for the control of COVID‐19 infection for patients, caregivers, and healthcare professionals. Moreover, it saves the cost of traveling and lodging near hospitals.^12^ The tele‐oncology platform is also a potential source of collaboration with specialists from other hospitals who are not immediately available for planning a personalized strategy for the patient.^13^ For these reasons, telemedicine shows economic and health advantages. The present study has two major unanswered questions: (1) the analysis of the economic impact; and (2) the detection rate of relapses with this follow‐up method. However, they do not represent the objectives of the investigation. Communication composed only of electronic tools may be perceived by patients and healthcare professionals as less empathetic in comparison with personal contact. Patients with cognitive disabilities may not be best suited for any assessment by telemedicine but, at the same time, may benefit by avoiding unnecessary travel.^14^ It is believed that this is the first and largest study involving telemedicine to follow up patients with female cancers during the COVID‐19 pandemic.

## 5 CONCLUSION

Telemedicine was generally well accepted in both cancer groups, not only among younger and higher‐educated women but even by women needing intensive care.

Although there are many points that can be improved, telemedicine is undoubtedly a tool that can also be used in the post–COVID‐19 era, thanks to its varying potential.

## CONFLICTS OF INTEREST

The authors have no conflicts of interest.

## AUTHOR CONTRIBUTIONS

EP and MM: substantial contributions to the conception or design of the work; CA, FG, and AM: acquisition, analysis, or interpretation of data for the work; AS, MGB, and SD: drafting the work or revising it critically for important intellectual content. All authors approved the final version to be published.
